# Supplementary material for: Validation of Multiplex PCR and Serology Detecting Helicobacter Species in Mice
Source: Microorganisms. 2023 Jan 18;11(2):249. doi: 10.3390/microorganisms11020249 (PMC9963394; doi:10.3390/microorganisms11020249)
Supplement: Supplementary file 1 [file microorganisms-11-00249-s001.zip › microorganisms-2120458-supplementary.pdf]

Supplement

Supplementary Table S1: *H. hepaticus* and *H. bilis* proteins included in multiplex serology and their amino acid identities with proteins of related *Helicobacter* species

			Amino acid identity (%)[a] to proteins of			
**Protein**	**Uniprot ID**	**Predicted function**	***H. hepaticus***	***H. bilis***	***H. typhlonius***	***H. pylori***
H. hepaticus (ATCC51449)						
HH1201	Q7U317	GroEL	Ref	83	93	84
HH0407	Q93PJ5	UreaseA	Ref	78	-	68
HH0713	Q7VI95	Lipoprotein	Ref	54	78	53
HH0966	Q7VHK1	-	Ref	31	75	49
HH1446	Q9RFY6	CdtA	Ref	43	60	-
HH0435	Q9X691	OmpA-like	Ref	49	79	49
HH0243	Q7VJK0	-	Ref	92	-	-
H. bilis (ATCC43879/WiWa)						
HRAG_00845	C3XFJ9	GroEL	83	Ref	82	82
HRAG_01470	C3XHC4	UreaseA	78	Ref	-	68
HRAG_01504	C3XHF8	Lipoprotein	57	Ref	55	54
HRAG_01298	C3XGV2	-	43	Ref	46	-
HRAG_01818	C3XIE3	CdtA	44	Ref	47	-
P167C	Q71SN5	-	-	Ref	-	-
P167D	Q71SN5	-	-	Ref	-	-

[a]as determined by BlastP[31]; -, No homologous protein found

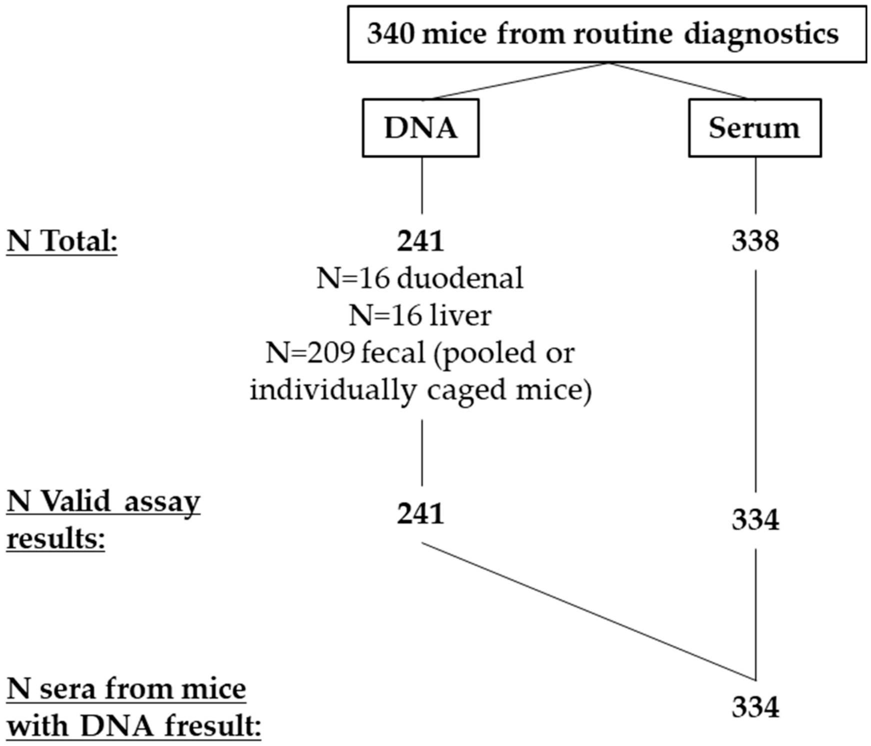

Supplementary Figure S1: Murine samples to assess sensitivity and specificity of the newly developed Helicobacter multiplex DNA finder and Helicobacter multiplex serology. Samples from n= 340 colony animals and sentinel mice (different transgenic strains, CD1, BL/6, NMRI and Mastomys coucha), which were tested in the frame of the routine health monitoring of rodents at the DKFZ, were used to assess sensitivity and specificity of the newly developed Helicobacter multiplex DNA finder and Helicobacter multiplex serology. The samples for PCR analysis comprised duodenal and liver samples from n=16 mice, as well as n=209 fecal samples (n=109 from individual mice and n=100 pooled fecal samples from two to three mice). Concurrent serum samples were available from n=338 mice (Figure 1). Valid PCR and serology assay results were available for n=241 and n=334 of the samples, respectively, resulting in concurrent PCR and serology data for n=334 mice.

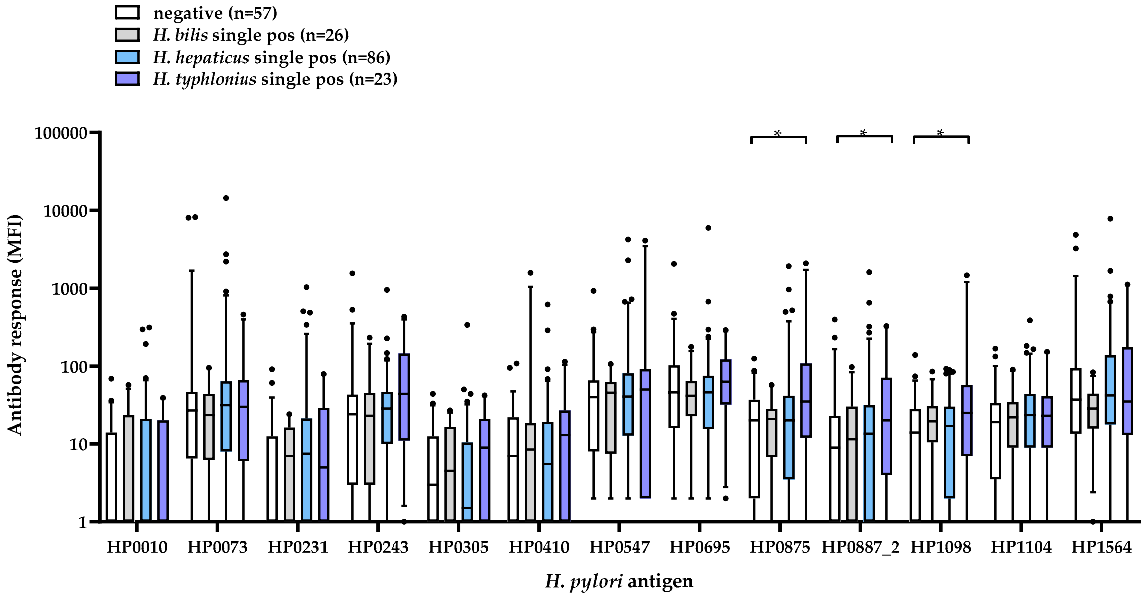

Supplementary Figure S2: Antibody responses [MFI] to *H. pylori* antigens by Helicobacter multiplex DNA finder result in murine samples taken at routine diagnostics. Boxes represent 25th to 75th and whiskers the 5th to 95th percentile, solid lines show the median. Dots represent data points lying outside the 5th and 95th percentiles, respectively. Wilcoxon-Mann-Whitney test was applied to compare continuous antibody responses [MFI] individual DNA-positive groups to the DNA-negative group: *p-value < 0.05
